# Machine learning improves 10-year prediction of atherosclerotic plaque progression and cardiovascular events in systemic lupus erythematosus

**DOI:** 10.1093/rheumatology/keag607

**Published:** 2026-10-29

**Authors:** Nikolaos Papazoglou, Petros P Sfikakis, Maria G Tektonidou

**Affiliations:** Rheumatology Unit, First Department of Propaedeutic Internal Medicine, Joint Academic Rheumatology Program, School of Medicine, National and Kapodistrian University of Athens, Athens, Greece; Rheumatology Unit, First Department of Propaedeutic Internal Medicine, Joint Academic Rheumatology Program, School of Medicine, National and Kapodistrian University of Athens, Athens, Greece; Rheumatology Unit, First Department of Propaedeutic Internal Medicine, Joint Academic Rheumatology Program, School of Medicine, National and Kapodistrian University of Athens, Athens, Greece

**Keywords:** systemic lupus erythematosus, cardiovascular risk prediction, cardiovascular disease, atherosclerotic plaques, vascular ultrasound, cardiovascular risk stratification, machine learning model

## Abstract

**Objectives:**

To evaluate the performance of generic and disease-adapted tools in predicting 10-year cardiovascular disease (CVD) events and atherosclerotic plaque progression risk in systemic lupus erythematosus (SLE), and develop a machine learning–based model to improve cardiovascular risk stratification.

**Methods:**

In a prospective 10-year follow-up carotid and femoral ultrasound study of patients with SLE without prior CVD events, we assessed the performance of 11 CVD risk scores (7 conventional, 4 SLE-adapted) to predict atherosclerotic plaque progression and incident CVD events. We used discrimination [sensitivity, specificity, area under the curve (AUC), Youden index], calibration (Brier Score, BS) and classification (Matthews correlation coefficient (MCC), F1 score) metrics, and logistic regression and time-to-event analyses. A machine learning–based gradient boosting model was developed and internally validated.

**Results:**

Among 111 patients (91% female, median age: 43.0 years) with 10-year ultrasonography follow-up, 53 (47.7%) developed new plaques, and 8 (7.2%) had incident CVD events. All scores demonstrated suboptimal performance; sensitivity: 0.00–0.44, AUC 0.58–0.79, and BS: 0.44–0.71. In multivariable analysis, QRISK3 was the only tool independently associated with CVD events and/or plaque progression (HR: 1.87, *P* = 0.027). A machine learning–based score that integrates QRISK3 with disease-related risk factors (disease duration, cumulative glucocorticoid dose, DORIS achievement, antiphospholipid antibodies, hydroxychloroquine) and additional traditional CVD risk factors (physical activity, waist circumference), demonstrated significantly better performance compared with all evaluated scores.

**Conclusion:**

Conventional and SLE-adapted tools underestimate CVD events and atherosclerosis progression risk in SLE. A machine learning–based model incorporating disease-specific and traditional cardiovascular risk factors into QRISK3 improves CVD risk stratification.

*Rheumatology* key messages

- Eleven cardiovascular risk scores underperformed in predicting 10-year atherosclerotic plaque progression and events in SLE
- A machine learning–based model integrating QRISK3 with key disease-related and traditional risk factors improved prediction
- This model can improve risk stratification in individuals classified as low-risk by conventional scores, facilitating timely prevention

## Introduction

Cardiovascular disease (CVD) remains a leading cause of morbidity and mortality in systemic lupus erythematosus (SLE) [1, 2]. Despite improvements in disease management, individuals with SLE exhibit a 2- to 3-fold higher risk for myocardial infarction or stroke compared with the general population, especially among young adults [3, 4]. SLE is also characterized by premature and rapid accrual of subclinical atherosclerosis, which is 2.5- to 4-fold more prevalent in SLE compared with the general population, and comparable to disorders with established heightened CVD risk, including diabetes mellitus [5, 6].

Accurate cardiovascular risk prediction and stratification in SLE remains challenging [7–12]. Studies have shown that the conventional prediction models underestimate both clinical and subclinical CVD risk in SLE [10, 11, 13, 14]. These scores were developed for the general population and rely heavily on traditional risk factors such as (older) age, (male) sex, lipids and blood pressure. Notably, SLE predominantly affects women and mainly young individuals, while traditional risk factors related to SLE such as low physical activity (due to pain, fatigue or depression) [15] and increased waist circumference (due to glucocorticoids and sedentary lifestyle), are missing from conventional scores. In addition, CVD risk in SLE is not fully explained by traditional risk factors, and it is likely driven by chronic inflammation, endothelial dysfunction and impaired vascular repair, leading to plaque development [16]. Since generic risk scores lack weighting for disease-specific CVD risk factors (CVRFs) such as those reported by the EULAR recommendations for CVD risk management [disease activity, medications (glucocorticoids, hydroxychloroquine), and antiphospholipid antibodies (aPL)] [17], they frequently misclassify vulnerable SLE patients as ‘low risk’. Probably due to the above factors, the performance of modified and SLE-adapted tools that applied multipliers in the conventional risk scores (e.g. ×1.5 or ×2.0) or included only the diagnosis of SLE as an independent risk factor in their algorithm, remained suboptimal [10, 11, 13, 14, 18–20].

Most of the previous studies compared the performance of only a few generic scores, without including SLE-adapted risk scores, and they mainly assessed cardiovascular event outcomes using retrospective data [13, 14]. Increasing evidence has shown that machine learning–based CVD risk prediction models can outperform traditional tools in predicting CVD events and subclinical atherosclerosis progression in high-risk populations [21, 22]. However, their application in SLE remains largely unexplored.

In the present study, we evaluated the performance of multiple generic and SLE-adapted cardiovascular risk scores in predicting incident CVD events and new atherosclerotic plaque development in patients with SLE during a 10-year prospective follow-up. We further developed a machine learning–based prediction model by integrating key disease-related and traditional CVRFs into the best-performing risk score to improve CVD risk prediction in SLE.

## Methods

### Study population and design

One hundred fifteen patients who underwent a baseline carotid and femoral artery ultrasound were invited for 3-, 7- and 10-year ultrasound follow-up evaluations of atherosclerotic plaque progression [6, 23]. All participants fulfilled the 2012 Systemic Lupus International Collaborating Clinics (SLICC) classification criteria for SLE [24]. Patients with co-existing APS fulfilled the Sydney classification criteria [25]. Exclusion criteria at baseline were prior atherosclerotic CVD events, diabetes mellitus, active infection, malignancy and pregnancy.

At baseline and during the 3-, 7- and 10-year follow-up visits, detailed demographic, clinical, laboratory and treatment-related data were recorded. Traditional CVRFs included age, sex, smoking status (current and pack-years), arterial hypertension (defined as antihypertensive treatment and/or systolic blood pressure ≥140 mmHg or diastolic blood pressure ≥90 mmHg based on the average of three sequential measurements after at least 10 min of rest), lipid profile [total, low-density lipoprotein (LDL) and high-density lipoprotein cholesterol, and triglycerides], body mass index, waist circumference, family history of coronary artery disease and estimated glomerular filtration rate (eGFR). Use of CVD-related medications, including antihypertensives, lipid-lowering agents and antiplatelets, was also documented.

Disease-related characteristics included disease duration, disease activity assessed semiannually by the Systemic Lupus Erythematosus Disease Activity Index 2000 (SLEDAI-2K), and disease damage measured annually by the SLICC/American College of Rheumatology Damage Index. Serological parameters were measured semiannually and included anti–double-stranded DNA antibodies, complement C3 and C4 levels, and aPL (anticardiolipin antibodies, anti-β2 glycoprotein I antibodies and lupus anticoagulant). Persistent aPL positivity was defined as positivity at all follow-up assessments (3-, 7- and 10-year visits). The Lupus Low Disease Activity State (LLDAS) [26] and Definition of Remission in SLE (DORIS) [27] were assessed for each patient as well as their persistence in ≥50%, ≥75% or 100% of the follow-up duration, reported as LLDAS50, LLDAS75, LLDAS100 and DORIS50, DORIS75, DORIS100, respectively. Medications included: cumulative glucocorticoid dose (total prednisone dose received before the baseline visit and throughout follow-up), hydroxychloroquine (use at baseline, and consistent use throughout follow-up), immunosuppressives and biologic therapies.

All participants provided written informed consent, and the study protocol was approved by the Institutional Review Board of Laiko General Hospital (Scientific Council approval number 16506), in accordance with the Declaration of Helsinki.

### Vascular ultrasound assessment

All vascular ultrasound examinations were performed in our Cardiovascular Research laboratory by the same experienced operator. Subclinical atherosclerosis was assessed bilaterally in the common carotid arteries, carotid bulbs, internal carotid arteries and common femoral arteries using high-resolution B-mode ultrasound (Vivid 7 Pro, GE Healthcare) equipped with a high-frequency linear array transducer. Atherosclerotic plaques on ultrasound were defined according to the Mannheim consensus as focal structures encroaching into the arterial lumen by ≥0.5 mm, or ≥50% relative to the surrounding intima–media thickness, or demonstrating an intima–media thickness ≥1.5 mm [28].

### Outcomes

The study outcome was a hierarchical composite end point: atherosclerotic event and/or plaque progression. Incident atherosclerotic CVD events during the 10-year follow-up were defined as the first occurrence of any of the following: myocardial infarction, acute coronary syndrome, coronary or peripheral arterial revascularization procedures, ischaemic stroke, transient ischaemic attack, peripheral arterial disease or sudden cardiac death. In participants who did not experience CVD events during follow-up, the outcome was any new atherosclerotic plaques after the baseline in the examined arterial segments over the 3, 7 and 10-year follow-up. Follow-up duration was defined as the time from baseline to the first occurrence of either a CVD event or new atherosclerotic plaque, death, loss to follow-up or completion of the 10-year follow-up, whichever occurred first.

### Cardiovascular risk scores

Using baseline clinical and laboratory parameters, we calculated 11 CVD risk scores, including 7 generic and 4 SLE-adapted tools. Generic risk scores included the Systematic Coronary Risk Evaluation (SCORE) [29], SCORE2 [18], Framingham Risk Score (FRS) [30], Pooled Cohort Risk Equation (PCRE) [31], Globorisk [32], Prospective Cardiovascular Münster (PROCAM) [33], and the Predicting Risk of cardiovascular disease EVENTs (PREVENT) [34]. Modified or SLE-adapted scores included the modified SCORE (mSCORE) [29], modified FRS (mFRS) [19], QRESEARCH Cardiovascular Risk Algorithm version 3 (QRISK3) [20], and the SLE cardiovascular risk (SLECRISK) equation [35]. Risk scores were calculated according to their predefined eligibility age range. Participants younger than the minimum eligible age were classified as low-risk. High-risk classification was defined using score-specific risk thresholds: ≥5% for SCORE and mSCORE; ≥7.5% for <50-year-old and ≥10% for 50- to 69-year-old individuals for SCORE2; ≥10% for QRISK3 and Globorisk; and ≥20% for FRS, mFRS, PROCAM, PCRE, PREVENT and SLECRISK.

### Statistical analysis

Quantitative variables are presented as median (interquartile range) and categorical variables as *n* (%). Group comparisons were performed using χ^2^, Mann–Whitney *U* or Student’s *t*-tests, as appropriate, after assessment of normality with Shapiro–Wilk testing. Predictive performance of 11 cardiovascular risk scores for 10-year incident atherosclerotic CVD events and/or new plaque development was assessed using calibration, discrimination and classification metrics. Risk reclassification based on plaques was evaluated with McNemar’s test.

Logistic regression and interval-censored Weibull proportional hazards models were used to examine associations of risk scores and traditional and disease-related cardiovascular risk factors with outcomes. Variables for multivariable models were selected according to significance in univariate analysis and clinical relevance [1, 4, 5, 17, 23], avoiding collinearity with score components.

A gradient boosting model (GBM) [36] was developed using XGBoost [37], internally validated with repeated 5-fold cross-validation (20 repetitions), and compared with conventional and SLE-adapted scores using out-of-fold predictions and bootstrap-derived 95% confidence intervals.

Analyses were performed in R, with two-sided α = 0.05. Detailed statistical methods are provided in Supplementary Data S1.

## Results

### Patient characteristics

Among 115 SLE patients (all White) with a baseline evaluation, 111 patients with a 10-year vascular ultrasonography follow-up were included in the study (Supplementary Fig. S1, flowchart). Patient characteristics are presented in Table 1. The median age at baseline was 43.0 years (IQR 36.0–52.0), and 91.0% of patients were female. The median disease duration was 7.0 years (IQR 1.5–14.0). At baseline, 38 (34.2%) patients had positive aPL, while persistent aPL positivity during follow-up was observed in 28 (25.2%) patients. The median SLEDAI at baseline was 0 (IQR 0–4), and the median SLICC was 0 (IQR 0–1). Most patients had low disease activity throughout the 10-year follow-up period, with 85 (76.6%) individuals achieving LLDAS ≥75%, and 53 (47.7%) achieving DORIS remission ≥75% of follow-up.

**Table 1:** Baseline characteristics of SLE individuals stratified by baseline plaque presence and 10-year plaque progression or CVD events.

	All patients (*N* = 111)	With plaques at baseline (*N* = 28)	No plaques at baseline (*N* = 83)	*P* ^a^	With atherosclerotic CVD events or plaque progression (*N* = 59)	No atherosclerotic CVD events or plaque progression (*N* = 52)	*P* ^b^
White individuals, *n* (%)	111 (100.0)	28 (100.0)	83 (100.0)	NA	59 (100.0)	52 (100.0)	NA
Age (years)	43.0 (36.0–52.0)	55.5 (46.5–61.5)	39.0 (34.0–46.0)	**<0.001**	48.0 (42.0–57.0)	36.0 (30.5–42.0)	**<0.001**
Sex (female), *n* (%)	101 (91.0)	27 (96.4)	74 (89.2)	0.245	53 (89.8)	48 (92.3)	0.649
Systolic blood pressure (mmHg)	116.0 (109.0–124.0)	123.5 (113.5–134.0)	115.0 (107.0–122.0)	**0.015**	117.0 (113.0–128.0)	115.0 (107.0–120.5)	0.079
Diastolic blood pressure (mmHg)	71.0 (66.0–75.2)	71.5 (66.5–77.0)	70.0 (66.0–75.0)	0.434	73.0 (66.0–78.0)	69.0 (65.5–73.0)	**0.047**
Smoking current, *n* (%)	45 (40.5)	13 (46.4)	32 (38.6)	0.463	29 (49.2)	16 (30.8)	**0.049**
Family history of CAD, *n* (%)	16 (14.4)	3 (10.7)	13 (15.7)	0.519	7 (11.9)	9 (17.3)	0.415
Total cholesterol (mg/dL)	188.5 (170.8–223.8)	210.0 (179.5–230.0)	180.0 (169.0–212.0)	**0.046**	207.0 (178.0–237.0)	176.0 (157.5–197.0)	**<0.001**
LDL (mg/dL)	107.5 (92.0–132.5)	125.0 (105.5–142.5)	105.0 (89.0–126.0)	**0.007**	121.0 (100.0–141.0)	102.5 (87.5–120.5)	**0.008**
HDL (mg/dL)	59.0 (49.0–71.2)	61.0 (48.0–73.5)	58.0 (49.0–69.0)	0.482	60.0 (47.0–78.0)	56.0 (50.0–65.0)	0.213
Triglycerides (mg/dL)	93.0 (63.0–125.2)	105.0 (72.5–124.5)	89.0 (60.0–126.0)	0.333	102.0 (65.0–139.0)	93.0 (59.0–121.5)	0.192
Body mass index (kg/m^2^)	24.6 (21.3–28.4)	26.9 (22.9–30.0)	24.0 (20.8–26.6)	**0.047**	25.0 (21.4–30.0)	23.8 (21.0–26.5)	0.077
Waist circumference (cm)	81.0 (73.0–90.0)	86 (79.0–97.5)	78 (72–88)	**0.010**	82 (74–96)	80 (72–86)	0.090
eGFR (mL/min/1.73 m^2^)	111.0 (102.0–116.0)	100.3 (90.1–110.6)	108.5 (102.5–116.8)	**0.010**	107.0 (97.9–114.6)	108.6 (102.2–117.9)	0.073
Exercise (min/week)	60.0 (0.0–210.0)	30.0 (0.0–210.0)	90.0 (0.0–210.0)	0.536	60.0 (0.0–180.0)	120.0 (0.0–240.0)	0.109
Antihypertensives, *n* (%)	40 (36.0)	15 (53.6)	25 (30.1)	**0.025**	23 (39.0)	17 (32.7)	0.491
Lipid-lowering drugs at baseline, *n* (%)	10 (9.0)	5 (17.9)	5 (6.0)	0.059	7 (11.9)	3 (5.8)	0.263
Antiplatelets at baseline, *n* (%)	34 (30.6)	10 (35.7)	24 (28.9)	0.500	20 (33.9)	14 (26.9)	0.426
Disease duration (years)	7.0 (1.5–14.0)	9.5 (1.5–15.5)	6.0 (1.0–13.0)	0.334	9.0 (1.0–17.0)	4.5 (1.5–11.5)	0.066
Lupus nephritis, *n* (%)	31 (27.9)	5 (17.9)	26 (31.3)	0.170	13 (22.0)	18 (34.6)	0.140
Cumulative prednisone dose at baseline, g	4.9 (0.2–13.3)	3.3 (0.0–13.0)	5.1 (0.4–13.3)	0.690	4.3 (0.4–13.7)	5.5 (0.1–13.0)	0.868
Cumulative prednisone dose at 10-year follow-up, g	12.6 (2.8–27.5)	5.8 (0.7–24.3)	13.1 (4.2–28.9)	0.165	13.5 (2.0–29.3)	10.9 (3.0–23.6)	0.550
Hydroxychloroquine use at baseline, *n* (%)	74 (66.7)	22 (78.6)	52 (62.7)	0.122	40 (67.8)	34 (65.4)	0.788
Consistent hydroxychloroquine use during follow-up, *n* (%)	60 (54.1)	14 (50.0)	46 (55.4)	0.619	30 (50.8)	30 (57.7)	0.470
Immunosuppressives use at baseline, *n* (%)	44 (39.6)	9 (32.1)	35 (42.2)	0.348	21 (35.6)	23 (44.2)	0.353
SLEDAI at baseline	0 (0–4)	1 (0–4)	0 (0–4)	0.352	0 (0–2)	0 (0–4)	0.672
SLICC at baseline	0 (0–1)	0 (0–1)	0 (0–1)	0.224	0 (0–1)	0 (0–1)	0.834
LLDAS50, *n* (%)	97 (87.4)	24 (85.7)	73 (88.0)	0.754	56 (94.9)	41 (78.8)	**0.010**
LLDAS75, *n* (%)	85 (76.6)	24 (85.7)	61 (73.5)	0.187	48 (81.4)	37 (71.2)	0.205
LLDAS100, *n* (%)	43 (38.7)	8 (28.6)	35 (42.2)	0.202	18 (30.5)	25 (48.1)	0.058
DORIS50, *n* (%)	76 (68.5)	20 (71.4)	56 (67.5)	0.697	41 (69.5)	35 (67.3)	0.805
DORIS75, *n* (%)	53 (47.7)	11 (39.3)	42 (50.6)	0.300	25 (42.4)	28 (53.8)	0.227
DORIS100, *n* (%)	25 (22.5)	5 (17.9)	20 (24.1)	0.494	12 (20.3)	13 (25.0)	0.557
Antiphospholipid syndrome at baseline, *n* (%)	20 (18.0)	6 (21.4)	14 (16.9)	0.587	14 (23.7)	6 (11.5)	0.095
aPL positivity at baseline, *n* (%)	38 (34.2)	14 (50.0)	24 (28.9)	**0.042**	25 (42.4)	13 (25.0)	0.054
Persistent aPL positivity, *n* (%)	28 (25.2)	11 (39.3)	17 (20.5)	**0.048**	18 (30.5)	10 (19.2)	0.172
Persistently high aPL titers, *n* (%)	13 (11.7)	7 (25.0)	6 (7.2)	**0.011**	8 (13.6)	5 (9.6)	0.519
Persistent triple aPL positivity, *n* (%)	5 (4.5)	3 (10.7)	2 (2.4)	0.067	3 (5.1)	2 (3.9)	0.754
Persistent LA positivity, *n* (%)	6 (5.4)	3 (10.7)	3 (3.6)	0.151	4 (6.8)	2 (3.9)	0.495
Persistent aCL (IgM or IgG) positivity, *n* (%)	21 (18.9)	10 (35.7)	11 (13.3)	**0.009**	12 (20.3)	9 (17.3)	0.684
Persistent aCL (IgM) positivity, *n* (%)	12 (10.8)	5 (17.9)	7 (8.4)	0.165	6 (10.2)	6 (11.5)	0.817
Persistent aCL (IgG) positivity, *n* (%)	16 (14.4)	8 (28.6)	8 (9.6)	**0.014**	8 (13.6)	8 (15.4)	0.785
Persistent anti-β2 GPΙ (IgM or IgG) positivity, *n* (%)	20 (18.0)	9 (32.1)	11 (13.3)	**0.025**	13 (22.0)	7 (13.5)	0.241
Persistent anti-β2 GPΙ (IgM) positivity, *n* (%)	8 (7.2)	5 (17.9)	3 (3.6)	**0.012**	6 (10.2)	2 (3.8)	0.199
Persistent anti-β2 GPΙ (IgG) positivity, *n* (%)	15 (13.5)	6 (21.4)	9 (10.8)	0.157	9 (15.3)	6 (11.5)	0.568

Demographic and clinical characteristics refer to baseline unless stated otherwise; data are presented as median (interquartile range) unless alternately specified. Values in bold indicate statistical significance (*P* < 0.05).

a Differences between patients with atherosclerotic plaques at baseline vs. those without.

b Differences between patients with incident atherosclerotic CVD events or plaque progression during the 10-year follow-up vs. those without.

Abbreviations: aCL: anticardiolipin antibodies; anti-β2 GPI: anti-beta2 glycoprotein I antibodies; aPL: antiphospholipid antibodies; CAD: coronary artery disease; CVD: cardiovascular disease; DORIS100, DORIS75, DORIS50 = achieving definition of remission in SLE throughout/100%, 75% and 50% of the follow-up period, respectively; eGFR: estimated glomerular filtration rate; HDL: high-density lipoprotein; LA: lupus anticoagulant; LDL: low-density lipoprotein; LLDAS100, LLDAS75, LLDAS50 = lupus low disease activity state throughout/100%, 75% and 50% of the follow-up period, respectively; NA: not applicable; SLEDAI: systemic lupus erythematosus disease activity index; SLICC: systemic lupus international collaborating clinics.

### Vascular ultrasound data

At baseline, 28 (25.2%) patients had atherosclerotic plaques; 24 had carotid and 19 had femoral plaques. During the 10-year follow-up, 53 (47.7%) individuals developed new atherosclerotic plaques [carotid (*n* = 47), femoral (*n* = 20), carotid and femoral (*n* = 14) plaques] and 8 (7.2%) patients developed atherosclerotic CVD events [sudden cardiac death due to cardiac arrest (*n* = 2), acute coronary syndrome (*n* = 2), transient ischaemic attack (*n* = 2), peripheral artery disease (*n* = 2)]. In total, 59 (53.2%) patients developed incident atherosclerotic CVD events and/or plaque progression during the 10-year follow-up.

Patients with plaques at baseline were older and had higher total and LDL cholesterol, larger waist circumference, and lower eGFR levels than those without plaques (Table 1). Patients who developed atherosclerotic CVD events or plaque progression were older and had higher total and LDL cholesterol, higher diastolic BP, and more frequent current smoking. LLDAS ≥50% was less frequent among patients with atherosclerotic CVD events or plaque progression, while persistent aPL positivity was higher in patients with plaques at baseline.

### Performance of generic and SLE-adapted scores

At baseline, all generic and SLE-adapted risk scores were significantly higher in patients with atherosclerotic plaques compared with those without plaques (Supplementary Table S1; *P* ≤ 0.033). Additionally, most generic (FRS, PCRE, Globorisk, PROCAM, PREVENT) and SLE-adapted tools (mFRS, mSCORE and QRISK3) remained significantly higher among patients who developed incident atherosclerotic CVD events or plaque progression versus those without (Supplementary Table S1; *P* ≤ 0.003). Among patients who experienced incident atherosclerotic CVD events or plaque progression, reclassifying CVD risk based on plaque presence at baseline has increased the proportion of patients classified as high-risk by 15.3–35.6 percentage points across the examined risk scores. This corresponds to an ∼1.3- to 5-fold higher identification of individuals who subsequently developed atherosclerotic CVD events or plaque progression (Supplementary Table S2; *P* ≤ 0.013).

The performance of conventional and SLE-adapted tools in predicting incident atherosclerotic CVD events and atherosclerotic plaque progression over the 10-year follow-up in patients with SLE is depicted in Table 2. Discrimination ability, as assessed by AUC, ranged from poor to moderate across all models (0.58–0.79). Among SLE-adapted tools, QRISK3 showed moderate discrimination capacity (AUC 0.72), achieving the highest sensitivity (0.44) and an acceptable specificity (0.75). Although FRS, mFRS, Globorisk and PROCAM yielded slightly higher AUC values (0.76–0.79), these scores exhibited markedly lower sensitivity (0.02–0.24). QRISK3 demonstrated the best overall balance between false negatives and false positives (Youden index 0.19). In terms of classification metrics, QRISK3 yielded the highest F1 (0.53), while Globorisk and QRISK3 showed the best MCC (0.25 and 0.20, respectively). Calibration assessed by BS was most favourable for QRISK3 and mFRS (0.44 for both), although for mFRS this was largely driven by accurate identification of event-free individuals (high specificity), rather than effective prediction of those who developed atherosclerotic CVD events and plaque progression.

**Table 2:** Performance of generic and SLE-adapted cardiovascular risk scores in predicting 10-year plaque progression and/or CVD events in SLE individuals.

	Sensitivity	Specificity	F1	Youden index	MCC	AUC	Brier score
SCORE	0.07	0.94	0.12	0.01	0.02	0.66	0.71
mSCORE	0.10	0.92	0.17	0.02	0.04	0.71	0.71
SCORE2	0.27	0.88	0.40	0.16	0.20	0.61	0.68
FRS	0.02	0.98	0.03	0.00	−0.01	0.78	0.50
mFRS	0.19	0.92	0.30	0.11	0.16	0.79	0.44
QRISK3	0.44	0.75	0.53	0.19	0.20	0.72	0.44
PCRE	0.02	0.98	0.03	0.00	−0.01	0.70	0.60
Globorisk	0.24	0.94	0.37	0.18	0.25	0.76	0.56
PROCAM	0.03	1.00	0.07	0.03	0.13	0.78	0.49
PREVENT	0.00	1.00	NA	0.00	NA	0.78	0.51
SLECRISK	0.02	1.00	0.03	0.02	0.09	0.58	0.57

Abbreviations: CVD: cardiovascular disease; MCC: Matthews correlation coefficient; AUC: area under the receiver operating characteristic curve; FRS: Framingham Risk Score; Globorisk: Global Cardiovascular Risk Score; mFRS: modified FRS (applying a multiplication factor of 2.0 in FRS); mSCORE: modified SCORE (applying a multiplication factor of 1.5 in SCORE); NA: not applicable; PCRE: Pooled Cohort Risk Equation; PROCAM: Prospective Cardiovascular Münster Study risk score; PREVENT: Predicting Risk of Cardiovascular Disease Events score; QRISK3: QRESEARCH Risk Estimator version 3; SCORE: Systematic Coronary Risk Evaluation; SCORE2: Systematic Coronary Risk Evaluation 2; SLECRISK: Systemic Lupus Erythematosus Cardiovascular Risk Score.

In univariate logistic regression analysis, QRISK3 and Globorisk were the only CVD risk prediction tools significantly associated with atherosclerotic CVD events or plaque progression during the 10-year follow-up (Supplementary Table S3). In multivariable models, QRISK3 (Table 3, model A) maintained an independent association, whereas Globorisk did not (Table 3, model B). Disease duration was the only disease-related determinant in both models.

**Table 3:** Multivariable models for predictors of 10-year plaque progression and/or CVD events in SLE individuals.

	OR	95% CI	*P*
Model A			
Disease duration at baseline (years)	1.10	1.02–1.19	**0.014**
DORIS remission at baseline	1.37	0.55–3.41	0.497
Cumulative prednisone dose at baseline (g)	0.97	0.93–1.00	0.085
aPL positivity at baseline	1.64	0.64–4.22	0.302
QRISK3 (high-risk category)	2.76	1.06–7.17	**0.037**
Model B			
Disease duration at baseline (years)	1.09	1.01–1.18	**0.020**
DORIS remission at baseline	1.17	0.48–2.85	0.737
Cumulative prednisone dose at baseline (g)	0.97	0.94–1.01	0.142
aPL positivity at baseline	1.60	0.63–4.09	0.322
Globorisk (high-risk category)	3.41	0.87–13.34	0.078
	**HR**	**95% CI**	***P***
Model C			
Disease duration at baseline (years)	1.03	1.00–1.07	0.077
DORIS75	0.74	0.39–1.37	0.336
Persistent aPL positivity during follow-up	1.30	0.72–2.36	0.390
Cumulative prednisone dose at 10-year follow-up (g)	0.98	0.96–1.01	0.181
QRISK3 (high-risk category)	1.87	1.07–3.24	**0.027**
Model D			
Disease duration at baseline (years)	1.03	1.00–1.07	0.079
DORIS75	0.74	0.39–1.37	0.335
Persistent aPL positivity during follow-up	1.30	0.71–2.38	0.403
Cumulative prednisone dose at 10-year follow-up (g)	0.98	0.96–1.01	0.181
QRISK3 (high-risk category)	1.87	1.07–3.24	**0.027**
Consistent hydroxychloroquine use during follow-up	0.99	0.58–1.69	0.967

Bold values indicate statistical significance (*P* < 0.05).

Abbreviations: CI: confidence intervals; CVD: cardiovascular disease; DORIS75: achieving Definition of Remission in SLE for at least 75% of the follow-up period; Globorisk: Global Cardiovascular Risk Score; HR: hazards ratio; OR: odds ratio; QRISK3: QRESEARCH Risk Estimator version 3.

In univariate time-to-event analysis, QRISK3, Globorisk and SCORE2 were the only CVD risk prediction tools significantly associated with incident atherosclerotic CVD events and plaque progression over the 10-year follow-up (Supplementary Table S4). After adjustment for disease duration, cumulative glucocorticoid exposure, DORIS achievement ≥75%, and persistent aPL positivity during follow-up, only QRISK3 retained statistical significance in multivariable analyses (Table 3, model C). Further adjustment for consistent hydroxychloroquine use yielded similar results (Table 3, model D). The multivariable models including Globorisk and SCORE2 are presented in Supplementary Table S5.

Sensitivity analyses after excluding patients who had atherosclerotic plaques at the baseline showed similar results for performance (Supplementary Table S6) and time-to-event (Supplementary Tables S7 and S8) analyses of CVD events and/or new plaque development.

### Machine learning–based model performance

The newly developed machine learning–based GBM integrated QRISK3 (the best-performing risk score in multivariable analysis) with SLE-related and traditional CVRFs not incorporated into the original QRISK3 algorithm. Selected features include physical activity and waist circumference among the traditional CVRFs, as well as disease duration, cumulative glucocorticoid exposure, aPL positivity, DORIS achievement and hydroxychloroquine use among the disease-related CVRFs. The relative importance (%) of QRISK3 and the selected features reflecting their contribution to the GBM is shown in Fig. 1. The performance characteristics of GBM in predicting incident atherosclerotic CVD events and new plaque development over the 10-year follow-up are presented in Table 4. GBM showed significantly better sensitivity, Youden index, BS, MCC and F1 score compared with all generic and SLE-adapted risk scores, whereas differences in AUC were small and not statistically significant (Fig. 2 and Supplementary Table S9). Box plots comparing the performance of the GBM with traditional and SLE-adapted risk scores across performance metrics are shown in Supplementary Fig. S2.

**Figure 1:**
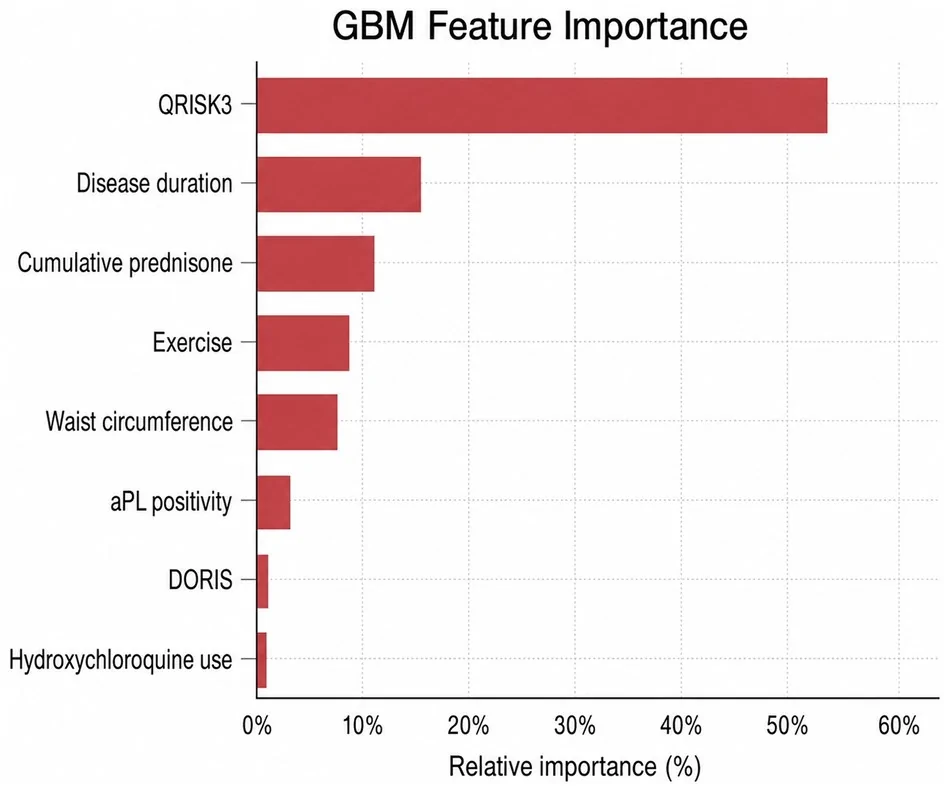
Relative importance (%) of QRISK3 and selected features reflecting their contribution in the GBM, as estimated by the XGBoost algorithm, for the prediction of incident atherosclerotic cardiovascular disease events and new plaque development over the 10-year follow-up in patients with SLE. aPL, antiphospholipid antibodies; DORIS, definition of remission in SLE; QRISK3, QRESEARCH Risk Estimator version 3

**Figure 2:**
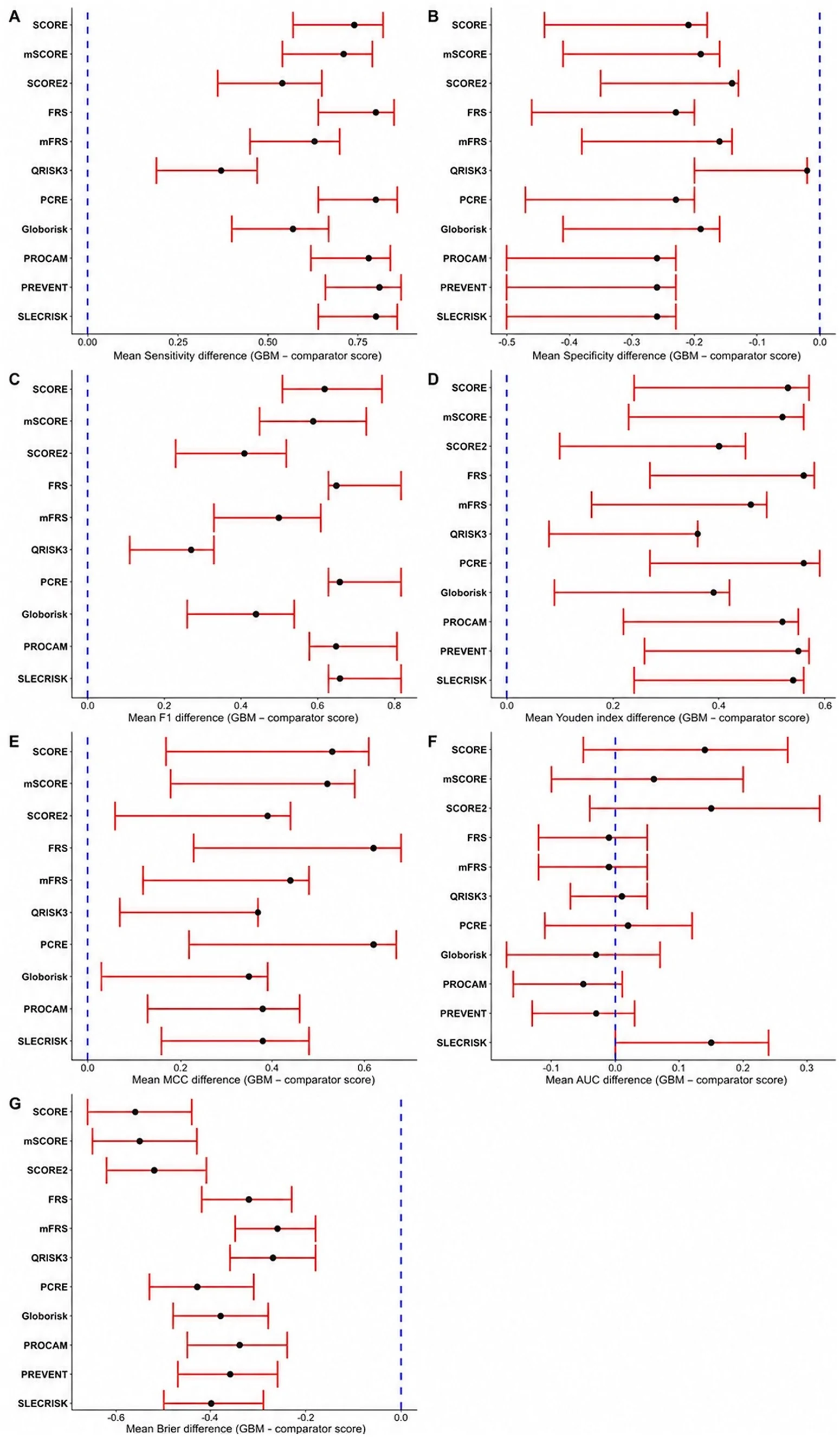
Mean differences (95% CI) in performance metrics between the GBM and conventional and SLE-adapted cardiovascular risk scores. Forest plots depicting mean differences (95% CI) in performance between the GBM and each risk score for the prediction of incident atherosclerotic cardiovascular events and new plaque development over 10 years in patients with SLE. Forest plots are shown for each performance metric: (A) sensitivity, (B) specificity, (C) F1 score, (D) Youden index, (E) MCC, (F) area under the receiver operating characteristic curve (AUC) and (G) Brier score. Points represent mean differences and horizontal lines indicate 95% confidence intervals derived from bootstrap resampling of out-of-fold predictions following 20× repeated 5-fold cross-validation. The vertical dashed line denotes zero (0); confidence intervals not crossing 0 indicate statistically significant differences. For sensitivity, specificity, F1 score, Youden index, MCC and AUC, positive values indicate higher values for the GBM than the comparator score. For Brier score, negative values indicate lower prediction error for the GBM

**Table 4:** Performance metrics of the GBM in predicting 10-year plaque progression and/or cardiovascular events in SLE individuals.

	Sensitivity	Specificity	F1	Youden index	MCC	AUC	Brier score
GBM	0.81	0.74	0.81	0.55	0.58	0.75	0.21

Abbreviations: MCC: Matthews correlation coefficient; AUC: area under the receiver operating characteristic curve. The model was developed using QRISK3 together with additional predictors measured at baseline, which included disease duration, cumulative glucocorticoids exposure, physical activity, waist circumference, antiphospholipid antibody positivity, the achievement of Definition of Remission in SLE (DORIS), and use of hydroxychloroquine.

## Discussion

Our 10-year longitudinal follow-up study showed that 11 evaluated cardiovascular risk prediction scores, including 7 conventional and 4 SLE-adapted tools, exhibited suboptimal performance in predicting 10-year incident atherosclerotic CVD events and subclinical atherosclerotic plaque progression in patients with SLE. Among SLE-adapted tools, QRISK3 was independently associated with incident atherosclerotic CVD events and new plaque development during the 10-year follow-up but with limited overall predictive performance. A newly developed machine learning–based model incorporating to QRISK3 the disease duration, cumulative glucocorticoid exposure, aPL positivity, DORIS achievement and hydroxychloroquine use along with physical activity and waist circumference, significantly improved its predictive performance compared with generic and other disease-adapted risk scores.

Growing evidence confirms that chronic, systemic inflammation and profound innate and adaptive immune dysregulation drive vascular damage and premature atherosclerosis [16] and arterial stiffness in SLE [38]. Although traditional CVRFs are more prevalent in SLE compared with the general population [39] and sustained CVRF control is associated with reduced clinical and subclinical atherosclerosis risk [23] and metabolic conditions in SLE [40], they do not fully explain the increased cardiovascular risk. In our cohort, both generic and SLE-adapted risk scores demonstrated suboptimal predictive performance. Their discrimination ranged from poor to moderate, with an overall limited ability to identify patients who developed atherosclerotic CVD events or atherosclerotic plaque progression during the 10-year follow-up. Our findings are consistent with previous studies, including those from our group, showing that both generic and SLE-adapted tools underestimate the burden of clinical and subclinical atherosclerotic CVD in patients with SLE [11, 41, 42]. In a prospective cohort study of 1887 SLE patients followed for 10 years, the FRS, SCORE and QRISK3 exhibited limited sensitivity in predicting incident cardiovascular events [41]. Similar findings have been reported in another prospective SLE cohort including 366 patients with a 10-year follow-up, where mFRS and mSCORE failed to adequately predict future CVD events [42]. Our group has previously shown that 5 generic and 3 SLE-adapted tools also underperformed in predicting 3-year subclinical atherosclerotic plaque progression in 124 individuals with SLE [11], underscoring their limited ability to detect early atherosclerotic disease in this population.

Among SLE-adapted risk prediction tools, QRISK3 demonstrated the best overall performance and emerged as the only score independently associated with incident atherosclerotic CVD events and atherosclerotic plaque progression in multivariable analyses. This finding is supported by another prospective study in SLE, in which QRISK3 demonstrated improved performance in predicting cardiovascular events compared with FRS and mFRS [42], and a previous study from our group, which showed that QRISK3, along with mFRS, was independently associated with 3-year plaque progression in patients with SLE [11]. In another longitudinal study, QRISK3 demonstrated comparable performance to mFRS in predicting cardiovascular events [41]. Divergent findings have been reported in retrospective studies. One study showed that QRISK3 improved the prediction of both clinical and subclinical atherosclerosis compared with the conventional tools SCORE2 and PREVENT [14], whereas another study demonstrated superior performance of mFRS over QRISK3, SLE cardiovascular risk equation (SLECRE), and the original FRS [13]. Although QRISK3 demonstrates a relative improvement over conventional and other SLE-adapted models, its predictive performance remains inconsistent across studies and may be further limited by a lack of validation outside the UK, particularly in multiethnic SLE populations [20].

In addition to traditional CVRFs, vascular ultrasound improves cardiovascular risk assessment. The 2021 ESC guidelines on CVD prevention considered the presence of atherosclerotic plaques on carotid ultrasound as a marker of very high CVD risk, irrespective of the estimated risk calculated by SCORE2 [18]. Importantly, this does not imply that asymptomatic plaques are equivalent to overt clinical CVD events, but rather that individuals with plaques warrant intensive CVRF management, similar to individuals with established clinical atherosclerotic CVD. In addition to predicting overt events, cardiovascular risk algorithms have also been significantly associated with atherosclerotic plaque presence [8], while the incorporation of measures of subclinical atherosclerosis into conventional models has been shown to improve risk prediction [43]. A general population study of 6961 individuals showed that the PREVENT score accurately predicted not only incident CVD events but also subclinical atherosclerosis [43]. In line with the above observations from the general population, we found that incorporating baseline plaque presence into risk assessment in patients with SLE significantly improved the prediction of 10-year incident atherosclerotic CVD events and/or plaque progression by ∼1.3- to 5-fold compared with prediction scores alone.

Previous efforts to develop SLE-adapted cardiovascular risk scores have been made [35, 44, 45]. The SLECRE [44], which was developed using Cox proportional hazards modelling, has demonstrated low specificity and poor discrimination compared with the FRS, mFRS and QRISK tools in two previous studies [13, 41]. The SLECRISK [35], derived using the least absolute shrinkage and selection operator (LASSO) with Cox regression, showed improved sensitivity for detection of coronary artery calcification compared with PCRE, however it remained suboptimal overall (30.8%) [46]. A systematic review and meta-analysis in the general population, including 32 machine learning and 26 conventional risk models, showed that machine learning–based algorithms performed better in predicting CVD events than the conventional models. This reflects their ability to capture complex, non-linear interactions among CVRFs not accounted for in traditional models [21]. To date, two studies have used machine learning methods in SLE for cardiovascular risk assessment [47, 48]; the first study evaluated the risk for cardiac involvement in general, without stratified results for atherosclerotic CVD events [47], while the second study estimated the stroke and myocardial infarction risk [48]. However, they had a different study design: both were retrospective studies, did not evaluate the risk of subclinical CVD (atherosclerotic plaque), and did not assess the performance of CVD risk prediction tools.

The systematic literature review that informed the 2022 EULAR recommendations for CVD management in rheumatic disorders, along with newer evidence, showed that disease duration, disease activity, glucocorticoids exposure and aPL are primary drivers of CVD risk in SLE, while hydroxychloroquine acts as a protective agent [17, 48–50]. Integrating the above disease-specific variables into the QRISK3 algorithm, along with physical activity and waist circumference, which are highly related to SLE traditional CVRFs, provides critical insights into cardiovascular risk prevention. Their inclusion resulted in significant improvement across most performance metrics (discrimination, classification and calibration) compared with all evaluated generic and SLE-adapted scores. In our cohort, baseline hydroxychloroquine use was common (66.7%) and comparable between individuals who developed incident CVD events and/or plaque progression and those who did not (67.8% vs. 65.4%, respectively; *P* = 0.788). Similarly, neither the baseline hydroxychloroquine use nor its consistent use during follow-up was significantly associated with incident CVD events and/or plaque progression in multivariable logistic regression and time-to-event analyses, respectively. The comparable baseline exposure and lack of a significant association with CVD events and/or plaque progression in multivariable models likely reduced the relative predictive contribution of hydroxychloroquine to the GBM. Furthermore, hydroxychloroquine use was incorporated into the model as a binary variable, which cannot capture treatment duration, adherence or cumulative exposure. This may be relevant, as growing evidence suggests that cardiovascular protection is more closely associated with long-term or sustained hydroxychloroquine exposure [17, 50].

Another important finding in our study was that patients with plaques at baseline and those who developed 10-year incident atherosclerotic CVD events or plaque progression had a higher CVRF burden and baseline risk score estimates. Sustained disease control, defined as LLDAS achievement over ≥50% of follow-up, was less frequently achieved in this group, while persistent aPL positivity was higher among individuals with plaques at baseline, further supporting the contribution of disease-related CVRFs to cardiovascular risk in SLE.

The strengths of our study include its prospective design, the comprehensive vascular ultrasound assessment in both carotid and femoral arteries, the use of predefined timepoints (baseline, 3, 7 and 10 years) and semi-annual laboratory/clinical feature assessments over the entire follow-up (e.g. assessment of LLDAS and DORIS 50%, 75% and 100%). We also developed for the first time a machine learning-based model for predicting the 10-year risk of both clinical and subclinical cardiovascular disease against various widely used generic and SLE-adapted risk scores. A potential limitation could be the relatively small number of patients (*n* = 111); however, the high percentage (95%) of patients at the baseline who retained a regular carotid/femoral ultrasound follow-up (at 3, 7 and 10 years) and a comprehensive semi-annual clinical and laboratory examination, allowed a reliable assessment of long-term outcomes and provided a robust dataset justifying the study’s statistical power. The prognostic value of femoral plaques in predicting CVD events is less well-established than that for prevalent carotid plaques; however, in our study only six individuals with new plaques developed isolated femoral plaques, indicating that our outcome was mainly driven by new carotid rather than isolated femoral plaque development. Plaque progression was defined as new plaque development in previously plaque-free arterial segments, rather than increasing intima-media thickness or enlargement of existing plaques. Since generic and disease-adapted CVD risk scores were originally developed to predict clinical CVD events rather than subclinical plaque progression, their underperformance may partly reflect the difference between their intended purpose and the composite outcome used in our study. Although internal validation was performed using repeated cross-validation to provide a reliable estimate of the model’s performance and generalizability, the proposed model should be interpreted in relation to the new development of both CVD events and subclinical atherosclerotic plaques rather than CVD events alone. Given the small number of incident CVD events (*n* = 8), its ability to predict CVD events alone requires validation in larger, multiethnic cohorts with a sufficient number of events before its clinical applicability can be confirmed.

In conclusion, widely used conventional and SLE-adapted cardiovascular risk prediction tools demonstrate suboptimal performance in identifying 10-year clinical and subclinical CVD risk in SLE. A machine learning–based model incorporating important disease-specific and traditional CVRFs into QRISK3 can enhance its predictive performance. Further validation in large, multi-ethnic populations is warranted to confirm the generalizability of these findings.

## Supplementary Material

keag607_Supplementary_Data

## Data Availability

Individual participant data from this study can be obtained from the corresponding author after de-identification upon reasonable request. Requests should be directed to mtektonidou@gmail.com or mtektonidou@med.uoa.g
