# Supplementary material for: Machine learning improves 10-year prediction of atherosclerotic plaque progression and cardiovascular events in systemic lupus erythematosus
Source: Rheumatology (Oxford). 2026 Oct 29;65(11):keag607. doi: 10.1093/rheumatology/keag607 (PMC13760283; doi:10.1093/rheumatology/keag607)
Supplement: Supplementary file 1 [file keag607_supplementary_data.docx]

Supplementary Material

**Machine learning improves 10-year prediction of atherosclerotic plaque progression and cardiovascular events in Systemic Lupus Erythematosus**

Nikolaos Papazoglou, MD^1^, Petros P. Sfikakis, MD, PhD^1^, and Maria G. Tektonidou, MD, PhD^1^

^1^Rheumatology Unit, First Department of Propaedeutic Internal Medicine, Joint Academic Rheumatology Program, School of Medicine, National and Kapodistrian University of Athens, Athens, Greece

**Supplementary Data S1: Methods**

Statistical Analysis

Quantitative variables were presented as median (interquartile range), and qualitative variables as frequencies (%). The Shapiro–Wilk test was used to assess data normality. Differences between groups were evaluated using the chi-square test, Mann–Whitney U test or Student’s t-test, as appropriate.

The performance of CVD risk scores in predicting 10-year incident atherosclerotic CVD events and atherosclerotic plaque progression was evaluated in terms of calibration (Brier Score [BS]), discrimination (sensitivity, specificity, area under the receiver operating characteristic curve [AUC], Youden index), and classification (Matthews correlation coefficient [MCC], F1 score) metrics. For metrics requiring binary risk classification into high- and low-risk categories (e.g., sensitivity, specificity), the predefined cut-off thresholds recommended by each risk score were applied. In individuals initially classified as low-risk, reclassification to the high-risk category was applied if atherosclerotic plaques were present at baseline ultrasound. Changes in high-risk classification before and after risk reclassification with plaques were evaluated using McNemar’s test.

Univariate logistic regression analyses examined potential determinants of 10-year incident atherosclerotic CVD events and new plaque development, including traditional and disease-related CVRFs, as well as all generic and SLE-adapted risk prediction scores at baseline, which were entered as binary covariates (yes/no) based on their respective high-risk classification thresholds. We included in the multivariate regression analysis variables with p<0.05 in univariate analysis, as well as the disease-specific CVRFs reported by the EULAR recommendations for CVD risk management in SLE, including disease duration, DORIS remission achievement, aPL positivity, cumulative corticosteroid exposure, and either QRISK3 or Globorisk at baseline [17]. To avoid collinearity, statistically significant parameters that were components of the evaluated risk scores were not included in the multivariable models.

As patients were reassessed at 3, 7, and 10 years after baseline, information on incident atherosclerotic CVD events or new plaque development was available within these follow-up intervals rather than only at the end of the 10-year follow-up. Because the exact timing of plaque development was not known and could only be determined to have occurred between consecutive visits (3-, 7-, and 10-year assessments), outcomes were treated as interval-censored. In time-to-event analysis, baseline generic and SLE-adapted risk prediction scores were used to quantify an individual's long-term hazard for developing cardiovascular events and/or new plaque development. We used univariate and multivariable parametric proportional hazards models with a Weibull baseline hazard, explicitly accounting for interval censoring. Covariate selection for multivariable models followed the same procedure applied in the logistic regression analyses.

No clinical, laboratory, or treatment-related data were missing for any completed study visit among patients with SLE included in the study; individuals with incomplete ultrasound follow-up assessments were included in the analyses and censored at their last available ultrasound assessment (Supplementary Figure S1).

To ensure that the presence of atherosclerotic plaques at baseline did not influence our findings, we conducted sensitivity analyses after excluding all patients with carotid and/or femoral plaques at baseline. These included a reassessment of the performance analysis for the different CVD risk scores, as well as time-to-event analyses for CVD events and/or new plaque development during the 10-year follow-up (Supplementary Tables S6-S8).

Machine learning-based prediction model

To further explore whether the performance of generic and/or SLE-adapted risk scores could be improved, we developed a machine learning-based gradient boosting model (GBM). Features in this model comprised baseline variables, including the best-performing risk score identified in our primary analysis (QRISK3, calculated using the publicly available online calculator, based on clinical and laboratory data from the baseline assessment), along with additional traditional and disease-related CVRFs not included in the original score algorithm. All variables included in the GBM were assessed at baseline; no prospectively collected follow-up data were used for the model development. Selected traditional CVRFs included physical activity, recorded as the average weekly duration of moderate-intensity exercise (minutes), and waist circumference, measured at the midpoint between the lower rib margin and the anterior superior iliac crest, in the standing position (centimeters). Disease-related features included disease duration from disease onset (years), cumulative glucocorticoid exposure until the baseline assessment, recorded as cumulative prednisone-equivalent dose (grams), DORIS remission status (yes/no), hydroxychloroquine use (yes/no), and aPL positivity, defined as positivity for at least one of lupus anticoagulant, anticardiolipin antibodies (IgG or IgM), or anti-β2-glycoprotein I antibodies (IgG or IgM), according to the Sydney criteria for classification of antiphospholipid syndrome. These additional factors were selected based on their clinical relevance as supported by the literature. The gradient boosting algorithm was implemented using XGBoost. As external validation data was not available, internal validation was performed using repeated 5-fold cross-validation (20 repetitions). Model performance was assessed on out-of-fold predictions at each iteration and summarized as the mean performance across all 100 validation runs. To compare the predictive performance of the new model with the generic and SLE-adapted risk scores, we also applied the latter to each out-of-fold dataset. For every out-of-fold dataset and each risk score, differences in all performance metrics of interest were calculated relative to the GBM. Mean differences were then summarized, and 95% confidence intervals were derived via 2000 bootstrap replications at the subject (ID) level of the stacked out-of-fold data.

All analyses were performed using R (R Foundation for Statistical Computing, Vienna, Austria). Statistical tests were two-sided and significance was set at α=0.05.

**Supplementary Figure S1**. Study flow-chart of individuals with SLE during the 10-year follow-up

Alt text: Flow chart showing screening, exclusions, and ultrasound follow-up availability of patients with SLE included in the 10-year study.

56 patients not included

34 excluded

24 due to prior clinical atherosclerotic cardiovascular disease

8 due to diabetes mellitus

2 due to malignancy

22 declined participation

171 consecutive patients with SLE screened

4 patients not eligible for follow-up examinations (excluded)

2 died before 3-year assessment

1 developed breast cancer

1 declined follow-up assessments

Baseline ultrasound examination

n=115 patients with SLE

10-year follow-up examination study

n=111 patients with SLE included

95 had a 10-year ultrasound assessment

9 had only a 3-year ultrasound assessment

7 had only 3- and 7-year ultrasound assessments

**Supplementary Table S1.** Median (IQR) values of generic and SLE-adapted cardiovascular risk scores with or without atherosclerotic plaques at baseline and with or without incident atherosclerotic CVD events or plaque progression during the 10-year follow-up

|  | Patients with SLE | With plaques at baseline | No plaques at baseline | P† | With CVD events or plaque progression | No CVD events or plaque progression | P‡ |
| --- | --- | --- | --- | --- | --- | --- | --- |
| SCORE | 0.4 (0.1–1.1), n=66 | 1.1 (0.4–1.3), n=25 | 0.2 (0.1–0.7), n=41 | **<0.001** | 0.7 (0.2–1.3), n=48 | 0.2 (0.1–1.0), n=18 | 0.050 |
| mSCORE | 0.6 (0.2–1.7), n=66 | 1.7 (0.6–2.0), n=25 | 0.3 (0.2–1.1), n=41 | **<0.001** | 1.1 (0.3–2.0), n=48 | 0.2 (0.2–0.9), n=18 | **0.009** |
| SCORE2 | 2.9 (1.3–4.1), n=66 | 3.9 (3.0–5.1), n=25 | 2.2 (1.1–3.2), n=41 | **<0.001** | 3.0 (1.8–4.1), n=48 | 1.8 (1.0–4.1), n=18 | 0.176 |
| FRS | 2.9 (1.5–6.2), n=105 | 5.4 (2.9–9.6), n=28 | 2.2 (1.3–4.5), n=77 | **0.001** | 4.8 (2.9–9.0), n=59 | 1.7 (0.9–2.6), n=46 | **<0.001** |
| mFRS | 5.8 (3.0–12.4), n=105 | 10.8 (5.8–19.1), n=28 | 4.4 (2.6–9.0), n=77 | **0.001** | 9.6 (5.8–18.0), n=59 | 3.4 (1.8–5.2), n=46 | **<0.001** |
| QRISK3 | 7.0 (3.3–13.2), n=108 | 11.6 (8.9–17.9), n=28 | 5.1 (2.8–11.7), n=80 | **<0.001** | 9.5 (6.0–15.7), n=59 | 4.1 (2.1–11.1), n=49 | **<0.001** |
| PCRE | 2.3 (0.9–4.3), n=80 | 4.6 (2.5–5.9), n=26 | 1.7 (0.6–2.9), n=54 | **<0.001** | 2.8 (1.8–4.6), n=52 | 1.1 (0.5–2.8), n=28 | **0.003** |
| Globorisk | 5.0 (3.0–9.0), n=79 | 8.0 (5.0–10.0), n=25 | 5.0 (2.0–8.0), n=54 | **0.002** | 7.0 (5.0–10.0), n=52 | 2.0 (2.0–5.0), n=27 | **<0.001** |
| PROCAM | 24.0 (13.0–33.0), n=111 | 35.5 (28.5–42.5), n=28 | 18.0 (10.0–29.0), n=83 | **<0.001** | 29.0 (23.0–38.0), n=59 | 14.0 (9.0–23.0), n=52 | **<0.001** |
| PREVENT | 1.0 (0.3–2.2), n=111 | 2.6 (1.5–3.4), n=28 | 0.7 (0.3–1.2), n=83 | **<0.001** | 1.5 (0.9–3.0), n=59 | 0.4 (0.2–0.9), n=52 | **<0.001** |
| SLECRISK | 4.6 (3.1–7.1), n=80 | 5.5 (4.0–8.5), n=26 | 4.0 (2.7–6.5), n=54 | **0.033** | 4.6 (3.6–7.4), n=52 | 4.5 (2.6–6.1), n=28 | 0.254 |

Data are presented as median (interquartile range). n represents the number of patients eligible for each risk score according to its predefined age range. Values in bold indicate statistical significance (p<0.05).

† Differences between patients with atherosclerotic plaques at baseline versus those without.
‡ Differences between patients with incident cardiovascular disease events or atherosclerotic plaque progression during the 10-year follow-up versus those without.

CVD: cardiovascular disease; SCORE: Systematic Coronary Risk Evaluation; mSCORE: modified SCORE (applying a multiplication factor of 1.5 in SCORE); SCORE2: Systematic Coronary Risk Evaluation 2; FRS: Framingham Risk Score; mFRS: modified FRS (applying a multiplication factor of 2.0 in FRS); QRISK3: QRESEARCH Risk Estimator version 3; PCRE: Pooled Cohort Risk Equation; Globorisk: Global Cardiovascular Risk Score; PROCAM: Prospective Cardiovascular Münster Study risk score; PREVENT: Predicting Risk of Cardiovascular Disease Events score; SLECRISK: Systemic Lupus Erythematosus Cardiovascular Risk Score; NA: not applicable

**Supplementary Table S2.** Ability of generic and SLE-adapted cardiovascular disease risk scores to identify patients with incident atherosclerotic CVD events or plaque progression during the 10-year follow-up (N=59) before and after risk reclassification based on plaque presence at baseline

|  | Patients classified as high-risk (N=59) | Patients classified as high-risk after risk reclassification with plaque presence (N=59) | Δ High-risk (percentage points) * | P-value † |
| --- | --- | --- | --- | --- |
| SCORE | 4 (6.8%) | 20 (33.9%) | +27.1 | **<0.001** |
| mSCORE | 6 (10.2%) | 21 (35.6%) | +25.4 | **<0.001** |
| SCORE2 | 16 (27.1%) | 26 (44.1%) | +16.9 | **0.013** |
| FRS | 1 (1.7%) | 18 (30.5%) | +28.8 | **<0.001** |
| mFRS | 11 (18.6%) | 23 (39.0%) | +20.3 | **<0.001** |
| QRISK3 | 26 (44.1%) | 35 (59.3%) | +15.3 | **0.004** |
| PCRE | 1 (1.7%) | 19 (32.2%) | +30.5 | **<0.001** |
| Globorisk | 14 (23.7%) | 27 (45.8%) | +22.0 | **<0.001** |
| PROCAM | 2 (3.4%) | 20 (33.9%) | +30.5 | **<0.001** |
| PREVENT | 0 (0.0%) | 21 (35.6%) | +35.6 | **<0.001** |
| SLECRISK | 1 (1.7%) | 19 (32.2%) | +30.5 | **<0.001** |

Data are presented as n (%). Risk classification refers to baseline estimates. Bold values indicate statistical significance (p<0.05).

* Δ indicates the absolute change (percentage points) in the proportion classified as high-risk before and after risk reclassification based on baseline plaque presence.

† P-values indicate changes in high-risk classification before versus after risk reclassification based on plaque presence within the group with incident atherosclerotic CVD events or plaque progression over the 10-year follow-up (McNemar test).

CVD: cardiovascular disease; SCORE: Systematic Coronary Risk Evaluation; mSCORE: modified SCORE (applying a multiplication factor of 1.5 in SCORE); SCORE2: Systematic Coronary Risk Evaluation 2; FRS: Framingham Risk Score; mFRS: modified FRS (applying a multiplication factor of 2.0 in FRS); QRISK3: QRESEARCH Risk Estimator version 3; PCRE: Pooled Cohort Risk Equation; Globorisk: Global Cardiovascular Risk Score; PROCAM: Prospective Cardiovascular Münster Study risk score; PREVENT: Predicting Risk of Cardiovascular Disease Events score; SLECRISK: Systemic Lupus Erythematosus Cardiovascular Risk Score

**Supplementary Table S3.** Univariate logistic regression analysis of incident atherosclerotic cardiovascular disease events or plaque progression during the 10-year follow-up

|  | **OR** | **95% CI** | **p-value** |
| --- | --- | --- | --- |
| Age (years) | 1.10 | 1.05–1.16 | **<0.001** |
| Sex (female), n (%) | 0.66 | 0.16–2.81 | 0.577 |
| Systolic blood pressure (mmHg) | 1.03 | 1.00–1.05 | 0.073 |
| Diastolic blood pressure (mmHg) | 1.05 | 1.00–1.10 | 0.055 |
| Family history of CAD, n (%) | 0.64 | 0.22–1.87 | 0.418 |
| Smoking current, n (%) | 2.50 | 1.08–5.78 | **0.033** |
| Total cholesterol (mg/dL) | 1.01 | 1.00–1.03 | **0.010** |
| LDL (mg/dL) | 1.01 | 1.00–1.02 | 0.096 |
| HDL (mg/dL) | 1.01 | 0.99–1.03 | 0.425 |
| Triglycerides (mg/dL) | 1.01 | 1.00–1.01 | 0.113 |
| Body mass index (kg/m²) | 1.07 | 0.99–1.16 | 0.080 |
| Waist circumference (cm) | 1.04 | 1.00–1.07 | **0.029** |
| eGFR (mL/min/1.73 m²) | 0.97 | 0.94–1.00 | 0.059 |
| Exercise (min/week) | 1.00 | 1.00–1.00 | 0.292 |
| Antihypertensives, n (%) | 1.47 | 0.64–3.40 | 0.362 |
| Lipid-lowering drugs, n (%) | 2.76 | 0.54–14.00 | 0.221 |
| Antiplatelets, n (%) | 1.23 | 0.52–2.90 | 0.642 |
| Disease duration (years) | 1.09 | 1.02–1.16 | **0.008** |
| Lupus nephritis, n (%) | 0.53 | 0.23–1.24 | 0.143 |
| SLEDAI | 1.02 | 0.91–1.14 | 0.721 |
| SLICC | 1.01 | 0.51–1.98 | 0.986 |
| Antiphospholipid syndrome, n (%) | 1.92 | 0.67–5.49 | 0.224 |
| Antiphospholipid antibody positivity, n (%) | 2.14 | 0.91–5.04 | 0.082 |
| Hydroxychloroquine use, n (%) | 1.13 | 0.49–2.59 | 0.777 |
| Immunosuppressives use, n (%) | 0.70 | 0.31–1.56 | 0.381 |
| Cumulative prednisone dose (g) | 1.00 | 0.97–1.03 | 0.874 |
| SCORE (high-risk), n (%) | 1.49 | 0.26–8.54 | 0.654 |
| mSCORE (high-risk), n (%) | 1.51 | 0.36–6.41 | 0.577 |
| SCORE2 (high-risk), n (%) | 2.83 | 0.95–8.45 | 0.063 |
| FRS (high-risk), n (%) | 0.72 | 0.04–11.91 | 0.821 |
| mFRS (high-risk), n (%) | 2.23 | 0.66–7.57 | 0.196 |
| QRISK3 (high-risk), n (%) | 2.60 | 1.08–6.23 | **0.032** |
| PCRE (high-risk), n (%) | 0.72 | 0.04–11.91 | 0.821 |
| Globorisk (high-risk), n (%) | 4.15 | 1.11–15.49 | **0.034** |
| PROCAM (high-risk), n (%) | NA | NA | NA |
| PREVENT (high-risk), n (%) | NA | NA | NA |
| SLECRISK (high-risk), n (%) | NA | NA | NA |

Variables refer to baseline; Values in bold indicate statistical significance (p<0.05).

OR: Odds Ratio; CI: confidence intervals; CAD: coronary artery disease; LDL: Low-Density Lipoprotein; HDL: High-Density Lipoprotein; eGFR: estimated Glomerular Filtration Rate; SLEDAI: Systemic Lupus Erythematosus Disease Activity Index; SLICC: Systemic Lupus International Collaborating Clinics; SCORE: Systematic Coronary Risk Evaluation; mSCORE: modified SCORE (applying a multiplication factor of 1.5 in SCORE); SCORE2: Systematic Coronary Risk Evaluation 2; FRS: Framingham Risk Score; mFRS: modified FRS (applying a multiplication factor of 2.0 in FRS); QRISK3: QRESEARCH Risk Estimator version 3; PCRE: Pooled Cohort Risk Equation; Globorisk: Global Cardiovascular Risk Score; PROCAM: Prospective Cardiovascular Münster Study risk score; PREVENT: Predicting Risk of Cardiovascular Disease Events score; SLECRISK: Systemic Lupus Erythematosus Cardiovascular Risk Score; NA: not applicable

**Supplementary Table S4.** Univariate time-to-event analysis of incident atherosclerotic cardiovascular disease events or plaque progression during the 10-year follow-up

|  | **HR** | **95% CI** | **p-value** |
| --- | --- | --- | --- |
| Age (years) | 1.04 | 1.02–1.06 | **<0.001** |
| Sex (female), n (%) | 0.72 | 0.31–1.69 | 0.450 |
| Systolic blood pressure (mmHg) | 1.02 | 1.00–1.03 | **0.025** |
| Diastolic blood pressure (mmHg) | 1.04 | 1.01–1.07 | **0.005** |
| Smoking current, n (%) | 1.67 | 1.00–2.80 | 0.050 |
| Family history of CAD, n (%) | 0.66 | 0.30–1.45 | 0.295 |
| Total cholesterol (mg/dL) | 1.01 | 1.00–1.01 | **0.009** |
| LDL (mg/dL) | 1.01 | 1.00–1.01 | 0.091 |
| HDL (mg/dL) | 1.00 | 0.99–1.01 | 0.511 |
| Triglycerides (mg/dL) | 1.00 | 1.00–1.01 | 0.060 |
| Body mass index (kg/m²) | 1.06 | 1.01–1.11 | **0.016** |
| Waist circumference (cm) | 1.03 | 1.01–1.05 | **0.003** |
| eGFR (mL/min/1.73 m²) | 0.99 | 0.97–1.00 | 0.079 |
| Exercise (min/week) | 1.00 | 1.00–1.00 | 0.143 |
| Antihypertensives, n (%) | 1.44 | 0.82–2.51 | 0.202 |
| Lipid-lowering drugs, n (%) | 1.42 | 0.57–3.58 | 0.452 |
| Antiplatelets, n (%) | 0.44 | 0.06–3.17 | 0.414 |
| Disease duration (years) | 1.05 | 1.01–1.08 | **0.008** |
| Lupus nephritis, n (%) | 0.74 | 0.40–1.37 | 0.337 |
| Cumulative prednisone dose at 10-year follow-up, g | 0.99 | 0.97–1.01 | 0.382 |
| Hydroxychloroquine use at baseline, n (%) | 1.17 | 0.67–2.02 | 0.583 |
| Consistent hydroxychloroquine use during follow-up, n (%) | 0.97 | 0.58–1.62 | 0.904 |
| Immunosuppressives use at baseline, n (%) | 0.79 | 0.47–1.36 | 0.400 |
| SLEDAI at baseline | 1.01 | 0.94–1.08 | 0.793 |
| SLICC at baseline | 0.95 | 0.62–1.46 | 0.822 |
| LLDAS50, n (%) | 1.85 | 0.58–5.94 | 0.299 |
| LLDAS75, n (%) | 1.11 | 0.56–2.20 | 0.765 |
| LLDAS100, n (%) | 0.60 | 0.35–1.04 | 0.070 |
| DORIS50, n (%) | 0.93 | 0.53–1.64 | 0.809 |
| DORIS75, n (%) | 0.78 | 0.47–1.32 | 0.363 |
| DORIS100, n (%) | 0.82 | 0.43–1.55 | 0.538 |
| Antiphospholipid syndrome at baseline, n (%) | 1.68 | 0.92–3.08 | 0.092 |
| Persistent aPL positivity during follow-up, n (%) | 1.42 | 0.81–2.48 | 0.215 |
| Persistently high aPL titers during follow-up, n (%) | 1.42 | 0.67–2.99 | 0.364 |
| Persistent triple aPL positivity during follow-up, n (%) | 1.01 | 0.32–3.24 | 0.981 |
| Persistent LA positivity during follow-up, n (%) | 1.14 | 0.41–3.14 | 0.804 |
| Persistent aCL (IgM or IgG) positivity during follow-up, n (%) | 1.17 | 0.62–2.20 | 0.635 |
| Persistent aCL (IgM) positivity during follow-up, n (%) | 1.01 | 0.44–2.36 | 0.973 |
| Persistent aCL (IgG) positivity during follow-up, n (%) | 0.99 | 0.47–2.10 | 0.987 |
| Persistent anti-β2 GPΙ (IgM or IgG) positivity during follow-up, n (%) | 1.30 | 0.70–2.41 | 0.411 |
| Persistent anti-β2 GPΙ (IgM) positivity during follow-up, n (%) | 1.87 | 0.80–4.37 | 0.149 |
| Persistent anti-β2 GPΙ (IgG) positivity during follow-up, n (%) | 1.05 | 0.52–2.15 | 0.884 |
| SCORE (high-risk) | 1.06 | 0.38–2.92 | 0.916 |
| mSCORE (high-risk) | 1.16 | 0.50–2.73 | 0.728 |
| SCORE2 (high-risk) | 1.79 | 1.00–3.19 | **0.048** |
| FRS (high-risk) | 0.69 | 0.10–5.06 | 0.719 |
| mFRS (high-risk) | 1.77 | 0.91–3.43 | 0.092 |
| QRISK3 (high-risk) | 1.89 | 1.13–3.17 | **0.016** |
| PCRE (high-risk) | 0.64 | 0.09–4.61 | 0.658 |
| Globorisk (high-risk) | 2.05 | 1.12–3.76 | **0.020** |
| PROCAM (high-risk) | 1.97 | 0.47–8.16 | 0.351 |
| PREVENT (high-risk) | NA | NA | NA |
| SLECRISK (high-risk) | 1.35 | 0.19–9.60 | 0.767 |

Variables refer to baseline unless stated otherwise. Values in bold indicate statistical significance (p<0.05).

CAD: coronary artery disease; LDL: Low-Density Lipoprotein; HDL: High-Density Lipoprotein; eGFR: estimated Glomerular Filtration Rate; SLEDAI: Systemic Lupus Erythematosus Disease Activity Index; SLICC: Systemic Lupus International Collaborating Clinics; LLDAS100, LLDAS75, LLDAS50: Lupus Low Disease Activity State throughout/100%, 75% and 50% of the follow-up period, respectively; DORIS100, DORIS75, DORIS50: achieving Definition of Remission in SLE throughout/100%, 75% and 50% of the follow-up period, respectively; aPL: antiphospholipid antibodies; LA: lupus anticoagulant; aCL: anticardiolipin antibodies; anti-β2 GPI: anti-beta2 glycoprotein I antibodies; SCORE: Systematic Coronary Risk Evaluation; mSCORE: modified SCORE (applying a multiplication factor of 1.5 in SCORE); SCORE2: Systematic Coronary Risk Evaluation 2; FRS: Framingham Risk Score; mFRS: modified FRS (applying a multiplication factor of 2.0 in FRS); QRISK3: QRESEARCH Risk Estimator version 3; PCRE: Pooled Cohort Risk Equation; Globorisk: Global Cardiovascular Risk Score; PROCAM: Prospective Cardiovascular Münster Study risk score; PREVENT: Predicting Risk of Cardiovascular Disease Events score; SLECRISK: Systemic Lupus Erythematosus Cardiovascular Risk Score; NA: not applicable

|  | **HR** | **95% CI** | **p-value** |
| --- | --- | --- | --- |
| **Model A** |  |  |  |
| Disease duration at baseline (years) | 1.04 | 1.00–1.08 | 0.058 |
| DORIS75 | 0.69 | 0.37–1.28 | 0.238 |
| Persistent aPL positivity during follow-up | 1.36 | 0.75–2.47 | 0.308 |
| Cumulative prednisone dose at 10-year follow-up (g) | 0.99 | 0.96–1.02 | 0.421 |
| Body mass index (kg/m²) | 1.06 | 1.01–1.12 | **0.021** |
| Globorisk (high-risk) | 1.42 | 0.70–2.88 | 0.332 |
|  |  |  |  |
| **Model B** |  |  |  |
| Disease duration at baseline (years) | 1.04 | 1.00–1.08 | **0.039** |
| DORIS75 | 0.67 | 0.36–1.25 | 0.208 |
| Persistent aPL positivity during follow-up | 1.32 | 0.73–2.39 | 0.355 |
| Cumulative prednisone dose at 10-year follow-up (g) | 0.99 | 0.96–1.02 | 0.487 |
| Body mass index (kg/m²) | 1.07 | 1.01–1.12 | **0.013** |
| SCORE2 (high-risk) | 1.4 | 0.75–2.60 | 0.292 |

**Supplementary Table S5.** Multivariable time-to-event models for predictors of incident atherosclerotic cardiovascular events and new atherosclerotic plaque development during the 10-year follow-up (including Globorisk and SCORE2)

Values in bold indicate statistical significance (p<0.05).

HR: Hazards Ratio; CI: confidence intervals; Globorisk: Global Cardiovascular Risk Score; SCORE2: Systematic Coronary Risk Evaluation 2

DORIS75: achieving Definition of Remission in SLE for at least 75% of the follow-up period.

**Supplementary Table S6.** Performance of generic and SLE-adapted cardiovascular risk scores in predicting 10-year plaque progression and/or cardiovascular events in the entire cohort of individuals with SLE and in the sensitivity analysis after excluding patients with plaques at baseline

|  | **Sensitivity** | **Specificity** | **F1** | **Youden Index** | **MCC** | **AUC** | **Brier Score** |
| --- | --- | --- | --- | --- | --- | --- | --- |
| SCORE | 0.07 → 0.03 (-0.04) | 0.94 → 0.98 (+0.04) | 0.12 → 0.05 (-0.07) | 0.01 → 0.00 (-0.01) | 0.02 → 0.01 (-0.01) | 0.66 → 0.77 (+0.11) | 0.71 → 0.70 (-0.01) |
| mSCORE | 0.10 → 0.05 (-0.05) | 0.92 → 0.98 (+0.06) | 0.17 → 0.10 (-0.07) | 0.02 → 0.03 (+0.01) | 0.04 → 0.08 (+0.04) | 0.71 → 0.79 (+0.08) | 0.71 → 0.69 (-0.02) |
| SCORE2 | 0.27 → 0.21 (-0.06) | 0.88 → 0.96 (+0.08) | 0.40 → 0.33 (-0.07) | 0.16 → 0.17 (+0.01) | 0.20 → 0.25 (+0.05) | 0.61 → 0.74 (+0.13) | 0.68 → 0.67 (-0.01) |
| FRS | 0.02 → 0.00 (-0.02) | 0.98 → 1.00 (+0.02) | 0.03 → NA | 0.00 → 0.00 (+0.00) | -0.01 → NA | 0.78 → 0.86 (+0.08) | 0.50 → 0.44 (-0.06) |
| mFRS | 0.19 → 0.13 (-0.06) | 0.92 → 0.98 (+0.06) | 0.30 → 0.23 (-0.07) | 0.11 → 0.11 (+0.00) | 0.16 → 0.21 (+0.05) | 0.79 → 0.87 (+0.08) | 0.44 → 0.39 (-0.05) |
| QRISK3 | 0.44 → 0.37 (-0.07) | 0.75 → 0.84 (+0.09) | 0.53 → 0.47 (-0.06) | 0.19 → 0.21 (+0.02) | 0.20 → 0.24 (+0.04) | 0.72 → 0.75 (+0.03) | 0.44 → 0.39 (-0.05) |
| PCRE | 0.02 → 0.00 (-0.02) | 0.98 → 1.00 (+0.02) | 0.03 → NA | 0.00 → 0.00 (+0.00) | -0.01 → NA | 0.70 → 0.79 (+0.09) | 0.60 → 0.55 (-0.05) |
| Globorisk | 0.24 → 0.18 (-0.06) | 0.94 → 0.98 (+0.04) | 0.37 → 0.30 (-0.07) | 0.18 → 0.16 (-0.02) | 0.25 → 0.27 (+0.02) | 0.76 → 0.84 (+0.08) | 0.56 → 0.51 (-0.05) |
| PROCAM | 0.03 → 0.03 (+0.00) | 1.00 → 1.00 (+0.00) | 0.07 → 0.05 (-0.02) | 0.03 → 0.03 (+0.00) | 0.13 → 0.12 (-0.01) | 0.78 → 0.80 (+0.02) | 0.49 → 0.43 (-0.06) |
| PREVENT | 0.00 → 0.00 (+0.00) | 1.00 → 1.00 (+0.00) | NA → NA | 0.00 → 0.00 (+0.00) | NA → NA | 0.78 → 0.82 (+0.04) | 0.51 → 0.44 (-0.07) |
| SLECRISK | 0.02 → 0.00 (-0.02) | 1.00 → 1.00 (+0.00) | 0.03 → NA | 0.02 → 0.00 (-0.02) | 0.09 → NA | 0.58 → 0.60 (+0.02) | 0.57 → 0.53 (-0.04) |

Values are shown as entire cohort → sensitivity analysis after excluding patients with baseline atherosclerotic plaques; the number in parentheses represents the absolute difference between the sensitivity analysis and the entire-cohort estimate

SCORE: Systematic Coronary Risk Evaluation; mSCORE: modified SCORE (applying a multiplication factor of 1.5 in SCORE); SCORE2: Systematic Coronary Risk Evaluation 2; FRS: Framingham Risk Score; mFRS: modified FRS (applying a multiplication factor of 2.0 in FRS); QRISK3: QRESEARCH Risk Estimator version 3; PCRE: Pooled Cohort Risk Equation; Globorisk: Global Cardiovascular Risk Score; PROCAM: Prospective Cardiovascular Münster Study risk score; PREVENT: Predicting Risk of Cardiovascular Disease Events score; SLECRISK: Systemic Lupus Erythematosus Cardiovascular Risk Score; NA: not applicable

**Supplementary Table S7.** Sensitivity univariate time-to-event analysis of 10-year plaque progression and/or cardiovascular events in individuals with SLE after excluding those with plaques at baseline

|  | **HR** | **95% CI** | **p-value** |
| --- | --- | --- | --- |
| Age (years) | 1.10 | 1.06–1.14 | **<0.001** |
| Sex (female), n (%) | 0.67 | 0.26–1.74 | 0.413 |
| Systolic blood pressure (mmHg) | 1.03 | 1.00–1.05 | **0.018** |
| Diastolic blood pressure (mmHg) | 1.04 | 1.00–1.07 | **0.041** |
| Smoking current, n (%) | 1.89 | 1.00–3.59 | 0.051 |
| Family history of CAD, n (%) | 0.91 | 0.38–2.19 | 0.837 |
| Total cholesterol (mg/dL) | 1.01 | 1.00–1.02 | **0.003** |
| LDL (mg/dL) | 1.01 | 1.00–1.02 | **0.035** |
| HDL (mg/dL) | 1.01 | 0.99–1.02 | 0.381 |
| Triglycerides (mg/dL) | 1.00 | 1.00–1.01 | **0.042** |
| Body mass index (kg/m²) | 1.07 | 1.01–1.13 | **0.021** |
| Waist circumference (cm) | 1.04 | 1.01–1.06 | **0.005** |
| eGFR (mL/min/1.73 m²) | 0.98 | 0.96–1.01 | 0.214 |
| Exercise (min/week) | 1.00 | 1.00–1.00 | 0.178 |
| Antihypertensives, n (%) | 1.19 | 0.60–2.36 | 0.625 |
| Lipid-lowering drugs, n (%) | 5.87 | 1.97–17.49 | **0.001** |
| Antiplatelets, n (%) | 1.00 | 0.49–2.01 | 0.995 |
| Disease duration (years) | 1.05 | 1.01–1.10 | **0.019** |
| Lupus nephritis, n (%) | 0.57 | 0.26–1.25 | 0.160 |
| Cumulative prednisone dose at 10-year follow-up, g | 1.00 | 0.98–1.01 | 0.753 |
| Hydroxychloroquine use at baseline, n (%) | 0.96 | 0.50–1.85 | 0.911 |
| Consistent hydroxychloroquine use during follow-up, n (%) | 0.99 | 0.52–1.87 | 0.965 |
| Immunosuppressives use at baseline, n (%) | 0.84 | 0.44–1.61 | 0.599 |
| SLEDAI at baseline | 1.00 | 0.93–1.09 | 0.923 |
| SLICC at baseline | 0.98 | 0.60–1.62 | 0.942 |
| LLDAS50, n (%) | 3.71 | 0.51–27.09 | 0.196 |
| LLDAS75, n (%) | 1.13 | 0.52–2.48 | 0.756 |
| LLDAS100, n (%) | 0.58 | 0.30–1.15 | 0.118 |
| DORIS50, n (%) | 0.79 | 0.40–1.55 | 0.492 |
| DORIS75, n (%) | 0.78 | 0.41–1.48 | 0.451 |
| DORIS100, n (%) | 0.99 | 0.47–2.09 | 0.976 |
| Antiphospholipid syndrome at baseline, n (%) | 2.00 | 0.94–4.26 | 0.071 |
| Persistent aPL positivity during follow-up, n (%) | 0.98 | 0.45–2.15 | 0.963 |
| Persistently high aPL titers during follow-up, n (%) | 0.61 | 0.15–2.55 | 0.500 |
| Persistent triple aPL positivity during follow-up, n (%) | 0.83 | 0.11–6.04 | 0.854 |
| Persistent LA positivity during follow-up, n (%) | 1.20 | 0.29–4.96 | 0.805 |
| Persistent aCL (IgM or IgG) positivity during follow-up, n (%) | 0.50 | 0.15–1.62 | 0.247 |
| Persistent aCL (IgM) positivity during follow-up, n (%) | 0.54 | 0.13–2.24 | 0.395 |
| Persistent aCL (IgG) positivity during follow-up, n (%) | 0.22 | 0.03–1.61 | 0.136 |
| Persistent anti-β2 GPΙ (IgM or IgG) positivity during follow-up, n (%) | 0.82 | 0.32–2.12 | 0.689 |
| Persistent anti-β2 GPΙ (IgM) positivity during follow-up, n (%) | 1.62 | 0.39–6.76 | 0.505 |
| Persistent anti-β2 GPΙ (IgG) positivity during follow-up, n (%) | 0.74 | 0.26–2.11 | 0.578 |
| SCORE (high-risk) | 1.59 | 0.22–11.68 | 0.650 |
| mSCORE (high-risk) | 2.37 | 0.55–10.17 | 0.246 |
| SCORE2 (high-risk) | 2.59 | 1.18–5.71 | **0.018** |
| FRS (high-risk) | NA | NA | NA |
| mFRS (high-risk) | 2.65 | 1.01–6.96 | **0.048** |
| QRISK3 (high-risk) | 2.41 | 1.24–4.69 | **0.010** |
| PCRE (high-risk) | NA | NA | NA |
| Globorisk (high-risk) | 3.06 | 1.32–7.10 | **0.009** |
| PROCAM (high-risk) | 4.50 | 0.54–37.45 | 0.164 |
| PREVENT (high-risk) | NA | NA | NA |
| SLECRISK (high-risk) | NA | NA | NA |

Variables refer to baseline unless stated otherwise. Values in bold indicate statistical significance (p<0.05).

HR: Hazards Ratio; CI: confidence intervals; CAD: coronary artery disease; LDL: Low-Density Lipoprotein; HDL: High-Density Lipoprotein; eGFR: estimated Glomerular Filtration Rate; SLEDAI: Systemic Lupus Erythematosus Disease Activity Index; SLICC: Systemic Lupus International Collaborating Clinics; LLDAS100, LLDAS75, LLDAS50: Lupus Low Disease Activity State throughout/100%, 75% and 50% of the follow-up period, respectively; DORIS100, DORIS75, DORIS50: achieving Definition of Remission in SLE throughout/100%, 75% and 50% of the follow-up period, respectively; aPL: antiphospholipid antibodies; LA: lupus anticoagulant; aCL: anticardiolipin antibodies; anti-β2 GPI: anti-beta2 glycoprotein I antibodies; SCORE: Systematic Coronary Risk Evaluation; mSCORE: modified SCORE (applying a multiplication factor of 1.5 in SCORE); SCORE2: Systematic Coronary Risk Evaluation 2; FRS: Framingham Risk Score; mFRS: modified FRS (applying a multiplication factor of 2.0 in FRS); QRISK3: QRESEARCH Risk Estimator version 3; PCRE: Pooled Cohort Risk Equation; Globorisk: Global Cardiovascular Risk Score; PROCAM: Prospective Cardiovascular Münster Study risk score; PREVENT: Predicting Risk of Cardiovascular Disease Events score; SLECRISK: Systemic Lupus Erythematosus Cardiovascular Risk Score; NA: not applicable.

**Supplementary Table S8.** Sensitivity multivariable time-to-event analysis models of 10-year plaque progression and/or cardiovascular events in individuals with SLE after excluding those with plaques at baseline

|  | **HR** | **95% CI** | **p-value** |
| --- | --- | --- | --- |
| **Model A** (including QRISK3) | | | |
| Disease duration at baseline (years) | 1.03 | 0.98–1.08 | 0.214 |
| DORIS75 | 0.76 | 0.34–1.70 | 0.500 |
| Persistent aPL positivity during follow-up | 0.91 | 0.40–2.04 | 0.817 |
| Cumulative prednisone dose at 10-year follow-up (g) | 0.98 | 0.96–1.01 | 0.305 |
| QRISK3 (high-risk category) | 2.18 | 1.03–4.64 | **0.042** |
| **Model B** (including SCORE2) | | | |
| Disease duration at baseline (years) | 1.03 | 0.99–1.09 | 0.174 |
| DORIS75 | 0.65 | 0.29–1.49 | 0.309 |
| Persistent aPL positivity during follow-up | 0.93 | 0.42–2.07 | 0.857 |
| Cumulative prednisone dose at 10-year follow-up (g) | 0.99 | 0.96–1.02 | 0.664 |
| Body mass index (kg/m²) | 1.07 | 1.01–1.15 | **0.029** |
| SCORE2 (high-risk category) | 1.83 | 0.75–4.45 | 0.181 |
| **Model C** (including mFRS) | | | |
| Disease duration at baseline (years) | 1.04 | 1.00–1.09 | 0.074 |
| DORIS75 | 0.71 | 0.31–1.60 | 0.406 |
| Persistent aPL positivity during follow-up | 0.96 | 0.42–2.17 | 0.923 |
| Cumulative prednisone dose at 10-year follow-up (g) | 1.00 | 0.97–1.03 | 0.763 |
| Body mass index (kg/m²) | 1.07 | 1.00–1.15 | **0.040** |
| mFRS (high-risk category) | 1.33 | 0.45–3.93 | 0.610 |
| **Model D** (including Globorisk) | | | |
| Disease duration at baseline (years) | 1.04 | 0.99–1.09 | 0.104 |
| DORIS75 | 0.71 | 0.31–1.61 | 0.416 |
| Persistent aPL positivity during follow-up | 1.01 | 0.45–2.30 | 0.978 |
| Cumulative prednisone dose at 10-year follow-up (g) | 0.99 | 0.96–1.02 | 0.689 |
| Body mass index (kg/m²) | 1.07 | 1.00–1.14 | 0.058 |
| Globorisk (high-risk category) | 1.86 | 0.73–4.74 | 0.194 |

Values in bold indicate statistical significance (p<0.05).

HR: Hazards Ratio; CI: confidence intervals; DORIS75: achieving Definition of Remission in SLE for at least 75% of the follow-up period; aPL: antiphospholipid antibodies; QRISK3: QRESEARCH Risk Estimator version 3; SCORE2: Systematic Coronary Risk Evaluation 2; mFRS: modified Framingham Risk Score; Globorisk: Global Cardiovascular Risk Score

**Supplementary Table S9.** Mean differences (95% CI) in performance metrics between the newly developed gradient boosting model and the traditional and SLE-adapted risk scores

|  | **Sensitivity difference (95% CI)** | **Specificity difference (95% CI)** | **F1 difference (95% CI)** | **Youden index difference (95% CI)** | **MCC difference (95% CI)** | **AUC difference (95% CI)** | **Brier score difference (95% CI)** |
| --- | --- | --- | --- | --- | --- | --- | --- |
| SCORE | **0.74 (0.57, 0.82)** | **-0.21 (-0.44, -0.18)** | **0.62 (0.51, 0.77)** | **0.53 (0.24, 0.57)** | **0.53 (0.17, 0.61)** | 0.14 (-0.05, 0.27) | **-0.56 (-0.66, -0.44)** |
| mSCORE | **0.71 (0.54, 0.79)** | **-0.19 (-0.41, -0.16)** | **0.59 (0.45, 0.73)** | **0.52 (0.23, 0.56)** | **0.52 (0.18, 0.58)** | 0.06 (-0.10, 0.20) | **-0.55 (-0.65, -0.43)** |
| SCORE2 | **0.54 (0.36, 0.65)** | **-0.14 (-0.35, -0.13)** | **0.41 (0.23, 0.52)** | **0.40 (0.10, 0.45)** | **0.39 (0.06, 0.44)** | 0.15 (-0.04, 0.32) | **-0.52 (-0.62, -0.41)** |
| FRS | **0.80 (0.64, 0.85)** | **-0.23 (-0.46, -0.20)** | **0.65 (0.63, 0.82)** | **0.56 (0.27, 0.58)** | **0.62 (0.23, 0.68)** | -0.01 (-0.12, 0.05) | **-0.32 (-0.42, -0.23)** |
| mFRS | **0.63 (0.45, 0.70)** | **-0.16 (-0.38, -0.14)** | **0.50 (0.33, 0.61)** | **0.46 (0.16, 0.49)** | **0.44 (0.12, 0.48)** | -0.01 (-0.12, 0.05) | **-0.26 (-0.35, -0.18)** |
| QRISK3 | **0.37 (0.19, 0.47)** | **-0.02 (-0.20, -0.02)** | **0.27 (0.11, 0.33)** | **0.22 (0.08, 0.36)** | **0.37 (0.07, 0.37)** | -0.01 (-0.07, 0.05) | **-0.27 (-0.36, -0.18)** |
| PCRE | **0.80 (0.64, 0.86)** | **-0.23 (-0.47, -0.20)** | **0.66 (0.63, 0.82)** | **0.56 (0.27, 0.59)** | **0.62 (0.22, 0.67)** | 0.02 (-0.11, 0.12) | **-0.43 (-0.53, -0.31)** |
| Globorisk | **0.57 (0.40, 0.67)** | **-0.19 (-0.41, -0.16)** | **0.44 (0.26, 0.54)** | **0.39 (0.09, 0.42)** | **0.35 (0.03, 0.39)** | -0.03 (-0.17, 0.07) | **-0.38 (-0.48, -0.28)** |
| PROCAM | **0.78 (0.62, 0.84)** | **-0.26 (-0.50, -0.23)** | **0.65 (0.58, 0.81)** | **0.52 (0.22, 0.55)** | **0.38 (0.13, 0.46)** | -0.05 (-0.16, 0.01) | **-0.34 (-0.45, -0.24)** |
| PREVENT | **0.81 (0.66, 0.87)** | **-0.26 (-0.50, -0.23)** | NA | **0.55 (0.26, 0.57)** | NA | -0.03 (-0.13, 0.03) | **-0.36 (-0.47, -0.26)** |
| SLECRISK | **0.80 (0.64, 0.86)** | **-0.26 (-0.50, -0.23)** | **0.66 (0.63, 0.82)** | **0.54 (0.24, 0.56)** | **0.38 (0.16, 0.48)** | 0.15 (0.00, 0.24) | **-0.40 (-0.50, -0.29)** |

Values represent mean differences (95% CI) in performance metrics between the gradient boosting model and each risk score for predicting incident atherosclerotic CVD events or new atherosclerotic plaque development over 10 years in patients with SLE, derived from bootstrap resampling of out-of-fold predictions following 20× repeated 5-fold cross-validation. Positive values indicate improved performance of the gradient boosting model compared with each risk score, whereas negative values indicate worse performance. Bold values indicate statistically significant differences (95% CI excluding zero)

CI: confidence intervals; MCC: Matthews correlation coefficient; AUC: area under the curve; SCORE: Systematic Coronary Risk Evaluation; mSCORE: modified SCORE (applying a multiplication factor of 1.5 in SCORE); SCORE2: Systematic Coronary Risk Evaluation 2; FRS: Framingham Risk Score; mFRS: modified FRS (applying a multiplication factor of 2.0 in FRS); QRISK3: QRESEARCH Risk Estimator version 3; PCRE: Pooled Cohort Risk Equation; Globorisk: Global Cardiovascular Risk Score; PROCAM: Prospective Cardiovascular Münster Study risk score; PREVENT: Predicting Risk of Cardiovascular Disease Events score; SLECRISK: Systemic Lupus Erythematosus Cardiovascular Risk Score; NA: not applicable

**Supplementary Figure S2.** Performance of the newly developed gradient boosting model and of traditional and SLE-adapted cardiovascular risk scores across different predictive performance metrics

Distribution of performance metrics across 20 times repeated 5-fold cross-validation runs for the gradient boosting model (XGB) and traditional and SLE-adapted cardiovascular risk scores for the prediction of incident atherosclerotic cardiovascular events and new plaque development over 10 years in patients with SLE. Panels display boxplots for (A) sensitivity, (B) specificity, (C) F1 score, (D) Youden index, (E) Matthews correlation coefficient, (F) area under the receiver operating characteristic curve (AUC), and (G) Brier score. Each boxplot summarizes the distribution of the corresponding performance metric across the 100 validation runs. The horizontal line represents the median, boxes indicate the interquartile range (IQR), whiskers extend to 1.5×IQR, points represent outliers, and crosses indicate mean values

Alt text: Boxplots showing the distribution of predictive performance metrics across repeated cross-validation runs for the gradient boosting model and conventional or SLE-adapted cardiovascular risk scores.
